# The Vitamin D–Folate Hypothesis as an Evolutionary Model for Skin Pigmentation: An Update and Integration of Current Ideas

**DOI:** 10.3390/nu10050554

**Published:** 2018-04-30

**Authors:** Patrice Jones, Mark Lucock, Martin Veysey, Emma Beckett

**Affiliations:** 1 School of Environmental & Life Sciences, University of Newcastle, Ourimbah, NSW 2258, Australia; patrice.jones@uon.edu.au (P.J.); emma.beckett@uon.edu.au (E.B.); 2 Hull-York Medical School, University of York, Heslington, York YO10 5DD, UK; martin.veysey@hyms.ac.uk; 3 School of Medicine and Public Health, University of Newcastle, Ourimbah, NSW 2258, Australia

**Keywords:** vitamin D, folate, skin pigmentation, ultraviolet radiation

## Abstract

Vitamin D is unique in being generated in our skin following ultraviolet radiation (UVR) exposure. Ongoing research into vitamin D must therefore always consider the influence of UVR on vitamin D processes. The close relationship between vitamin D and UVR forms the basis of the “vitamin D–folate hypothesis”, a popular theory for why human skin colour has evolved as an apparent adaption to UVR environments. Vitamin D and folate have disparate sensitivities to UVR; whilst vitamin D may be synthesised following UVR exposure, folate may be degraded. The vitamin D–folate hypothesis proposes that skin pigmentation has evolved as a balancing mechanism, maintaining levels of these vitamins. There are several alternative theories that counter the vitamin D–folate hypothesis. However, there is significant overlap between these theories and the now known actions of vitamin D and folate in the skin. The focus of this review is to present an update on the vitamin D–folate hypothesis by integrating these current theories and discussing new evidence that supports associations between vitamin D and folate genetics, UVR, and skin pigmentation. In light of recent human migrations and seasonality in disease, the need for ongoing research into potential UVR-responsive processes within the body is also discussed.

## 1. Introduction

Vitamin D is of ongoing research interest, stemming in part from our relatively recent discovery of vitamin D receptors (VDRs) in almost every system in the body. Vitamin D elicits the majority of its functions through binding to these receptors, which act as transcription factors that modulate gene expression by binding to vitamin D response elements on target genes [1]. The main physiological function of vitamin D is in regulating calcium levels by influencing calcium absorption, storage, and retention in the intestines, bones, and kidneys. Unsurprisingly, VDRs are ubiquitous at these sites [1]. VDRs are also found in a variety of other cell types, including the heart, skin, pancreas, parathyroid, and immune cells; it is estimated that ~5% of the human genome is under the influence of vitamin D [2,3,4,5].

Considerable research on vitamin D has focused on elucidating the non-calcemic roles of vitamin D and the extent to which VDRs influence human biology. Discovered roles involve the regulation of cell proliferation and differentiation, hormone secretion, and immune responses by vitamin D [6,7,8,9,10,11,12]. However, much remains to be elucidated about the vitamin D system. Notably, research must always consider that vitamin D is unique in being the only vitamin we produce endogenously in the skin following ultraviolet radiation (UVR) exposure [13]. Subsequently, vitamin D does not fit the classic definition of a vitamin, and it is often more accurately referred to as a pro-hormone or secosteroid. For most, the UVB-induced (280–315 nm) synthesis of vitamin D is the major source of this nutrient [1], but levels produced following UVR exposure are determined by several factors. Skin pigmentation is one of the greatest determinants of vitamin D production, given significant competition between melanin pigments and vitamin D precursors in absorbing UVB radiation [13,14,15].

The close connection of vitamin D with UVR and degree of pigmentation is the basis of the “vitamin D–folate hypothesis”, which aims to explain the evolution of human skin pigmentation [16]. Vitamin D and folate are two unrelated nutrients with disparate sensitivities to UVR; vitamin D is synthesised and folate is degraded in the skin following UVR exposure [1,17,18,19,20,21]. It is important to note here that vitamin D may also be degraded by UVR in cases of prolonged exposure; however, this is a proposed mechanism that serves to prevent vitamin D toxicity in high-UVR environments [22]. The unique UVR sensitivities of vitamin D and folate have led to the development of the vitamin D–folate hypothesis as a prominent theory explaining the apparent adaption of human skin colour to the UVR environment [16]. There are several alternative theories for the evolution of skin pigmentation [23,24]. However, there is evident overlap between these theories and the vitamin–D folate hypothesis. The focus of this review is to present an update on the vitamin D–folate hypothesis by integrating these theories and discussing new evidence that supports associations between vitamin D, folate, UVR, and skin pigmentation. 

## 2. The Vitamin D–Folate Hypothesis

The vitamin D–folate hypothesis is one of the main theories potentially explaining the evolution of human skin pigmentation. It is apparent that human skin tones arose as an adaptation to our UVR climate, with individuals of the darkest pigmentation having an origin in high-UVR areas near to the equator and lighter-skinned populations arising in low-UVR regions closer to the poles. This pattern arose as a result of two clines in skin pigmentation, whereby our early human ancestors firstly evolved to have darker skin pigmentation while residing in Africa but then evolved to lose their pigmentation when out-of-Africa migrations occurred [25]. The vitamin D–folate hypothesis proposes that these two clines of skin pigmentation evolved as a balancing mechanism to maintain levels of two vitamins: vitamin D and folate [16].

Vitamin D and folate are linked by their disparate sensitivities to UVR. UVR, on the one hand, stimulates the production of vitamin D in the skin, but, on the other hand, it may cause folate degradation through the absorption of UVR by folates or the oxidation of folates via free radicals following UVR exposure [1,17,18,19,20,21]. The vitamin D–folate hypothesis proposes that the original cline for increased pigmentation in high-UVR environments was driven by a need to protect folate levels against UVR-driven degradation. In turn, the second cline for depigmentation is suggested to have occurred to facilitate adequate vitamin D production in areas of lower UVR [16].

This hypothesis is plausible, given that in maintaining levels of vitamin D and folate via skin pigmentation processes, the widespread action of these nutrients in maintaining reproductive success would have been preserved [26,27]. The vitamin D status influences the reproductive health of both men and women and is associated with adverse pregnancy outcomes, semen quality, and the production of sex hormones [26]. Since the relatively recent discovery of VDRs in reproductive tissues of both sexes, studies on VDR-null mice link vitamin D inadequacy to a decline in sperm counts and motility, and aberrations in the testis, gonads, ovary, and uterus [26,28,29,30]. A link between the folate status and adverse pregnancy outcomes is well established, particularly with respect to the influence of this vitamin on the occurrence of neural tube defects [31]. Folate has importance in processes of DNA synthesis, repair, and methylation, and disruption to these processes can significantly impact on maternal and embryonic physiology [27] and affect paternal fertility by reducing sperm counts and motility [32]. The potential impacts of a deficiency of these nutrients on natural selection is an ongoing debate and is a common argument raised against the vitamin D–folate hypothesis. However, these arguments often do not consider that the benefits of an adequate vitamin D and folate status on reproductive success extend far beyond their roles in maintaining reproductive health. Vitamin D and folate regulate many mechanisms that offer immediate protection from potential lethal environmental stresses at life stages before reproduction. The following sections provide key examples of such mechanisms, which relate to other prominent theories for the evolution of skin pigmentation.

## 3. Alternative Theories for the Evolution of Skin Pigmentation

There are several alternative theories that counter the vitamin D–folate hypothesis. The skin mutagenesis and skin barrier hypotheses are two prominent theories proposed as explanations for the evolution of darker skin pigmentation [23,24], and the energy conservation hypothesis is an alternative theory put forth to explain subsequent depigmentation [23]. It is important to clarify that these are not alternative hypotheses to the vitamin D–folate hypothesis per se, as there is significant overlap between these theories and the actions of vitamin D and folate in the skin. The evidence for these theories afford explanatory power; however, it remains the case that these hypotheses relate to thousands of years of environmentally driven adaptive pressures, and such paradigms are difficult to test within contemporary populations. The manifestation of several theories for the evolution of skin colour is unsurprising. Skin colour is such an extensive polygenic adaptation to our environment that the explanation for its evolution is likely a complex picture that integrates these prominent theories and current unknowns.

### 3.1. The Skin Mutagenesis Hypothesis

The skin mutagenesis theory proposes that skin pigmentation arose as a mechanism to protect against the development of skin cancers [23]. This hypothesis is based on the observations that more highly pigmented individuals are at lower risks of developing skin cancers because of the ability of skin pigmentation to combat UVR impact [33]. It stands to reason that the pigment-facilitated protection of the vitamin D and folate status is involved in this hypothesis, as vitamin D and folate exert several photoprotective actions that would combat against skin malignancies [34,35,36,37,38,39,40,41]. For example, vitamin D reduces UVR-induced DNA damage and cell death via an influence on multiple cell-cycle regulators (e.g., proto-oncogenes and tumour suppressors) and levels of reactive oxygen species [34,35,36,37,38,39]. The actions of folate in DNA synthesis and repair pathways are important mechanisms to repair UVR-induced DNA damage and maintain genomic integrity [40,41]. This hypothesis has several limitations that affect plausibility. Notably, there is much skepticism, given that the most fatal forms of skin cancer peak after reproductive age, and it is therefore difficult to argue that a low occurrence of skin cancers in individuals of reproductive age alone would have had an impact on natural selection [42,43]. However, it is proposed that a selective advantage of protecting older (post-reproductive) adults against skin malignancies can be seen when considering the importance of older generations for offspring survival in hunter–gatherer communities [44,45].

### 3.2. The Skin Barrier Hypothesis

The skin barrier hypothesis proposes that highly pigmented skin arose as a barrier that protected against multiple environmental stresses [23]. UVR exposure causes deleterious changes in skin morphology, which reduces the ability of the skin to act as a defense barrier. Such damage includes disruption to skin permeability and a subsequent increase in transepidermal water loss [46]. The skin barrier hypothesis is based on evidence that darker-pigmented skin types possess an enhanced barrier function compared to lighter skin types, mainly attributed to the role of melanin in scattering UVR [23,47]. Compared to lighter skin types, darkly pigmented skin is shown to possess more robust permeability and greater structural integrity, barrier recovery, and skin surface acidity [23,47]. In areas of high UVR and extreme humidity, darker skin pigmentation would have protected against the disruption of skin permeability via UVR and subsequent excessive water losses, and the acidity of the skin would have acted as a defense mechanism against microbial invasions. However, the feasibility of this theory is potentially limited, given that considerable discrepancies exist between studies examining ethnic differences in skin morphology.

This hypothesis is proposed as a discrete theory to the vitamin D–folate hypothesis. However, vitamin D and folate exert an array of functions that regulate the skin as a barrier against environmental stresses, having roles not only in the development of skin structures, but also in defense mechanisms that protect against UVR, heat, and microbial stresses.

The role of vitamin D in the skin is an area that has received significant research. The skin is a hub of vitamin D activity, as skin keratinocytes are unique in being the only cells in the body capable of both producing and metabolising vitamin D; being our primary site of vitamin D_3_ synthesis and also possessing all enzymes needed to metabolise inert vitamin D to its active form, calcitriol [48]. Vitamin D, in turn, regulates several pathways involved in maintaining skin integrity.

Vitamin D regulates many processes involved in the development of the stratum corneum, the outmost layer of our skin. This is a highly permeable layer that expresses multiple elements of our adaptive and innate immune system, operating as a barrier against extensive water loss and microbial invasion [13]. Vitamin D promotes the differentiation of keratinocytes into cells of the stratum corneum (corneocytes) via modulating calcium levels and the expression of protein components of the skin [13]. Vitamin D also regulates the permeability of this layer via involvement of VDR in the synthesis of long-chain glycosylceramides, which form part of the lipid-enriched membranes around corneocytes [49]. In addition, this nutrient plays several roles in immune responses in the skin. As examples, vitamin D may increase the expression of a key antimicrobial protein, cathelicidin, and the secretion of cytokines from T-cells: two modes of antimicrobial defense expressed on the skin surface [50]. Folate may also play a part in skin immune responses, although this role is not well understood. Folate deficiency is associated with a decline in cell-mediated immunity, driven by a reduction in T-cell proliferation [51]. The folate status is also linked to the expression of multiple proteins involved in immune function, inflammation, and coagulation in human blood [52]. Notably, a high folate status correlates with increases in the expression of proteins involved in the activation and regulation of the complement system, an important non-specific skin defense mechanism [52].

Folate may have a role in melanogenesis by regulating the production and stabilisation of tetrahydrobiopterin [53,54,55,56]. Tetrahydrobiopterin is a required cofactor for tyrosine hydroxylase, which converts tyrosine into dopa in the production of melanin pigments [57]. It could be suggested that folate and melanin compounds are synergistic; melanin, on the one hand, protects folate from UVR-related degradation, which in turn supports the influence of folate in melanogenesis. Interestingly, tetrahydrobiopterin also acts as a cofactor in the synthesis of nitric oxide, which has its primary function as a vasodilator in blood vessels [54,55,56]. Vasodilation is the body’s primary response to heat stress, with increased blood flow allowing body heat to be lost via the skin through convection [58]. From an evolutionary perspective, our ability to maintain vasodilation/vasoconstriction mechanisms would have been important in surviving varying UVR environments. As these mechanisms may been seen as relatively short-term responses to temperature changes, they are likely to be of greater importance in temperate UVR environments rather than environments of high UVR. This is supported by nitric oxide dependent vasodilation shown to be reduced in darkly skinned populations [59]. This suggests that vasodilation processes offer no advantage in extreme UVR environments but may be important in temperate UVR environments, where seasonal and daily temperature fluctuations are seen. Vitamin D is also suggested to influence vasodilation by its influence on nitric oxide synthase [60] and vasoconstriction by influences on the renin-angiotensin system [61].

### 3.3. The Metabolic Conservation Hypothesis

The metabolic conservation theory proposes that the depigmentation of ancestral humans can be explained by a need to draw resources away from melanin production and towards other metabolic processes [23]. Melanogenesis involves several production steps and feedback/crosstalk mechanisms that are dependent on energy input [62]. In migrating to lower-UVR environments, it is likely that we lost the pressure to produce melanin to counter UVR-related stresses. The metabolic conservation theory suggests that the evolution of intermediate European and Asian skin tones allowed for the shunting of resources away from melanin production, to be used instead to combat against stressors associated with colder climates. However, this theory still supports the likeliness of the extreme dilution of pigmentation amongst northern Europeans being a mechanism to facilitate vitamin D production in low-UVR environments [23]. 

The conservation theory is therefore not entirely isolated from the vitamin D–folate hypothesis. It is not a stretch to suggest that the protection of the vitamin D and folate status via pigmentation acted as the predominant pressure for the evolution of contrasting skin types at the equator and near the poles, as the likelihood of a deficiency in these nutrients would be highest in these environments. The occurrence of intermediate skin types that display facultative pigmentation in central European and Asian populations would have allowed for adequate vitamin D production. However, the primary “driver” may have been a need to restrict melanin production and channel these resources into responding to increased energy needs associated with colder climates. However, this is not to say the importance of vitamin D in intermediate UVR environments would have been obsolete. Notably, functions of vitamin D and folate in vasodilation/vasoconstriction outlined above, as well as roles in adipocyte biology, may have been important in maintaining energy and temperature homeostasis in increasingly colder climates.

Energy stored in adipose tissue can be utilised to maintain cellular functions in cases of increased energy needs or to fuel adaptive thermogenesis in response to cold stress [63]. When energy is scarce, energy needs can be generated in white adipose tissue via an increase in fatty acid β-oxidation and the subsequent shunting of fatty acids into the electron transport chain to generate adenosine triphosphate (ATP). A similar mechanism occurs in brown adipose tissue, which has a principal role in regulating adaptive thermogenesis. In brown adipose tissue, β-oxidation results in the generation of energy in the form of heat [63].

The roles of vitamin D in regulating fatty acid β-oxidation, energy metabolism, and the formation of brown adipose tissue are indicated by studies employing VDR-null mice models [63,64]. These actions are proposed to involve the role of vitamin D in regulating the expression of related genes, with the vitamin D status being associated with the expression of genes such as *PGC1α*, *PPARα*, *UCP1*, *SIRT1*, and *AMPK* involved in mitochondrial biogenesis and thermoregulation [65]. The roles of vitamin D in adipose tissue may have been important in increasingly colder climates. Even in temperate climates, these mechanisms would have been needed to respond to daily and seasonal variations in temperature and subsequent changes in energy needs [58]. Notably, this theory is supported by evident ethnic differences in cold responses, with darkly skinned subjects being more susceptible to cold injury compared to lighter-skinned individuals [66,67,68]. It could therefore be suggested that the occurrence of depigmentation in areas of lower UVR was a necessary measure to not only preserve energy but also allow for more efficient responses to colder regimes.

## 4. New Evidence Supporting the Vitamin D–Folate Hypothesis

The vitamin D–folate hypothesis was first proposed by Branda and Eaton in the 1970s [69]. This hypothesis has been further developed by Jablonski and Chaplin in more recent years [70]. One question that remained when the vitamin D–folate hypothesis was first refined was whether the distribution of genetic variants involved in vitamin D and folate processes is related to the UVR environment [25]. In such a case, the integration of vitamin D and folate genes with UVR would provide a strong argument for the involvement of these nutrients in the evolution of skin pigmentation. Much research has emerged since providing support for this. 

Apparent differences are consistently reported between the frequency of common *VDR* variants and ethnicity [71]. For examples, the common *VDR* variant *Fok1* has a lower frequency in African populations compared to European/Asian populations [71], with the frequency of another variant, *Cdx2,* being the highest in African populations and the lowest in Europeans [72]. These ethnic differences in the allele frequency likely reflect an adaption to different UVR regimes. This is supported by a recent study reporting multiple loci involving the *VDR* gene that display strong latitudinal clines [73] and previous work of the present authors in showing positive associations between the carriage of several *VDR* variant alleles (*Fok1*, *Bsm1*, *Apa1*, and *Taq1*) and latitude (as a surrogate measure of UVR exposure) [74]. Ancestral alleles for *Bsm1*, *Fok1,* and *Taq1* variants are associated to increases in cytokine production [75], immune cell response [76], and *VDR* expression [77], respectively. The carriage of these alleles is the highest in lower latitudes (i.e., areas associated with high UVR levels), indicating the selection of *VDR* genotypes that facilitated vitamin D modulated immune responses in these environments. Conversely, the occurrence of variant alleles for *Apa1*, *Fok1*, and *Bsm1* is increased at higher latitudes (associated with lower UVR levels) and is associated with increases in serum vitamin D levels [78,79]. In these environments, an increase in circulatory vitamin D may be important in responding to increased energy needs and cold stress.

Multiple studies support the idea that genetic variation in vitamin D metabolism genes influences the vitamin D status [80]. Notably, interactions are observed between UVR exposure and genetic variants in the vitamin D metabolism genes *CYP2R1* and *GC* for predicting UVB-induced vitamin D concentrations [81]. Similarly, common *GC* variants are reported to account for 10% of racial differences in circulating vitamin D levels [82]. These genetic determinants are suggested to lead to differences in the bioavailability of circulating vitamin D and account for differences in the vitamin D status between ethnicities. It has been argued that these variants, therefore, argue against the vitamin D–folate hypothesis and are adaptive measures to overcome skin pigmentation as a barrier for adequate vitamin D synthesis. However, the existence of UVR-responsive vitamin D genes is consistent with the vitamin D hypothesis, and the amount of variation in the vitamin D status that can be explained by these genetic factors is small (1–10%) compared to other factors such as skin pigmentation [81,82]. 

Several relationships are reported between UVR and folate metabolism genes [83,84,85]. The activity of serine hydroxymethyltransferase (SHMT) and methylene tetrahydrofolate reductase (MTHFR) enzymes is shown to be UVR responsive, with the translation of SHMT shown to increase in cells exposed to UVR [83] and a significant negative association observed between UVR exposure and frequency of the *MTHFR*-C677T variant, which results in a thermo-labile form of MTHFR [84]. The present author and colleagues have also shown an association between the latitude and frequency of several polymorphisms in folate genes (*MTHFR*-C677T, *MTHFR*-A1298C, *TYMS* 28bp 2R>3R, and *SHMT*-C1420T) [85]. More recently, the present authors have also reported significant associations between the frequency of 16 common folate variants and the degree of skin pigmentation via an analysis of genotypic data from over 30,000 individuals from different global populations [86]. These studies indicate trends between gene variant frequency and skin pigmentation that may occur in a manner that limits the incidence of genotypes that may adversely influence folate metabolism, particularly in populations of darker skin types residing in areas of high UVR levels. As a key example, the incidence of the *MTHFR*-C677T variant, closely linked to aberrant folate-dependent processes, is the lowest in darker-skinned populations [86]. Collectively, these findings indicate the existence of interactions between UVR, skin type, and vitamin D and folate genes, and they provide supporting molecular evidence for the vitamin D–folate hypothesis.

## 5. Integrating Current Theories

The explanation for the evolution of skin colour is likely a complex integration of our current predominant theories (vitamin D–folate, skin mutagenesis, skin barrier, and energy conservation hypotheses). This review provides key examples of overlap between the theories proposed as alternatives to the vitamin D–folate hypothesis and actions of vitamin D and folate in countering environmental stress.

**Figure 1:**
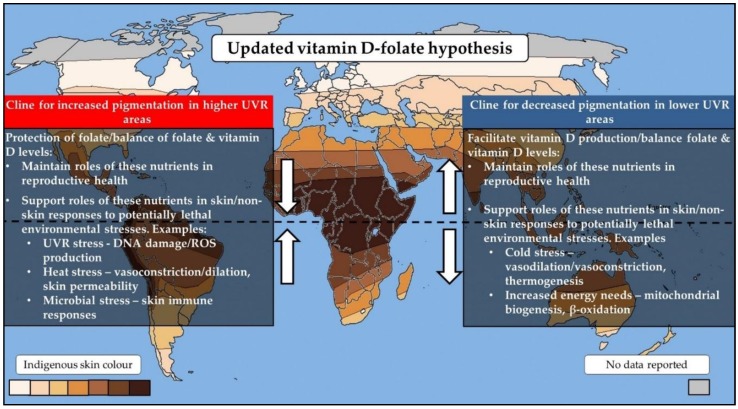
An update of the vitamin D–folate hypothesis. Vitamin D and folate have disparate sensitivities to UVR; whilst vitamin D may be synthesised following UVR exposure, folate may be degraded. The vitamin D–folate hypothesis proposes that the two clines of skin pigmentation evolved as a balancing mechanism to maintain levels of these photosensitive vitamins. In maintaining adequate levels of vitamin D and folate, roles of these nutrients in reproductive health would be preserved. Protection of vitamin D and folate levels may have offered additional advantages in the form of these nutrients themselves having roles in maintaining the skin as a barrier against environmental stresses. Vitamin D also exerts roles in adipocytes that may be of importance in colder environments. These additional roles are consistent with precepts of other prominent theories for the evolution of skin pigmentation (skin mutagenesis, skin barrier, and energy conservation hypotheses). UVR: ultraviolet radiation; ROS: reactive oxygen species. Map adapted from Chaplin (2004) [87].

The vitamin D–folate paradigm proposes that skin pigmentation evolved as a balancing mechanism to maintain levels of two key vitamins in human health: vitamin D and folate. In maintaining levels of these vitamins, the roles of these nutrients during reproduction would be preserved. This review discusses a likely mutually beneficial relationship between vitamin D, folate, and skin pigmentation that updates and further extends the vitamin D–folate hypothesis (Figure 1). The protection of levels of vitamin D and folate via skin pigmentation may have offered an additional advantage, because these nutrients themselves have roles in maintaining the skin as a barrier against environmental stresses. Vitamin D also has roles in adipocytes that may be of potential importance in increasingly colder, generally low UVR environments. These roles are consistent with precepts of other theories for the evolution of skin pigmentation, and they support the integration of these theories. Emerging research supports the interaction of genes involved in vitamin D and folate processes with UVR and skin pigmentation, providing the most recent support for the vitamin D–folate hypothesis.

## 6. Relevance to Public Health

The vitamin D–folate hypothesis and related theories propose that skin pigmentation evolved to regulate the biological effect of differing UVR levels in different regions of the globe [10]. However, the migration of human populations across large distances over the last several hundred years has created an evident mismatch between the adapted skin types of individuals and their UVR environment, a common characteristic of Westernised countries, where residents live in an environment receiving UVR levels drastically different from their ancestral areas [43].

A significant motivator for understanding the relationship between skin pigmentation and UVR-responsive processes is the potential health consequences of this mismatch. As a consequence of migration patterns, many darkly pigmented individuals now reside in areas of low UVR, and individuals with lighter skin tones are exposed to heightened levels of UVR [43]. These individuals are at risk of UVR-adaptive mechanisms being shifted, either leading to the risk of an inadequate vitamin D–folate status or the risk of having inadequate skin defense mechanisms for a specific environment.

The current recommendations for vitamin D and folate are based on requirements needed to prevent deficiency diseases. However, our greatest motivator to further examine the relationship between UVR and vitamin D and folate systems is perhaps the accumulating evidence linking these vitamins to the risk and onset of many current chronic diseases, such as cardiovascular diseases, diabetes, and several cancers [88,89]. Notably, there is considerable interest, in particular, on the role of vitamin D in reducing the risk for such chronic diseases, but these associations remain controversial [90,91]. Several of these chronic health outcomes also display seasonality, indicating that there is a potential interaction between UVR and vitamin D–folate in the etiology of such outcomes that may influence differences in study outcomes [92,93,94,95,96,97,98]. In better understanding the extent to which UVR, vitamin D, and folate interact, we may better understand how these factors may interact to influence human health and disease.
